# PP28 Leveraging Social Media For Educating Laypeople On Health Technology Assessment: The Participa SUS/ATS Experience

**DOI:** 10.1017/S026646232510247X

**Published:** 2025-12-29

**Authors:** Quenia Cristina Dias Morais, Dominique Spada, Andressa Braga, Milene Rangel da Costa, Bruno Barros, Marisa Santos

## Abstract

**Introduction:**

Understanding health technology assessment (HTA) is essential for effective public participation in Brazil’s Unified Health System (SUS). The Participa SUS/ATS project simplifies the concepts of HTA to foster training and knowledge dissemination. Through scientific marketing on social media, it provides interactive posts, making HTA accessible and empowering lay audiences to actively engage in decision-making processes.

**Methods:**

The project leveraged social media platforms (LinkedIn, YouTube, and Instagram) to disseminate knowledge on HTA and train lay audiences of the SUS. Educational videos, including versions with Brazilian Sign Language, illustrations for inclusivity, whiteboards, and live sessions carried out by the project team and guests addressing topics of social participation, were shared on YouTube. A logo was created as part of its visual identity. Instagram featured weekly polls, stories, and personalized posts using accessible and engaging language. LinkedIn promoted broad professional dialogue. This multiplatform approach combined multiple formats to increase reach and understanding, and to promote public interaction and empowerment in HTA processes.

**Results:**

By 2024, the initiative garnered 518 Instagram followers, 218 YouTube subscribers, and 1,202 LinkedIn followers. LinkedIn promoted conversations among healthcare professionals, Instagram’s engaging weekly posts connected with audiences, and the most-watched YouTube video garnered 323 views. Real-time interaction was encouraged with three live sessions. A specialized logo improved brand recognition and connection with the intended audience. Carousel postings were a user-friendly format, particularly on mobile devices. Users praised the content’s clarity and emphasized how well it demystified HTA contents. Posting schedule optimization raised exposure and interaction. Feedback highlighted improved understanding, empowering users to contribute more effectively to decision-making processes.

**Conclusions:**

The Participa SUS/ATS project showcases an innovative use of social media to demystify HTA for diverse audiences, fostering inclusivity and active public engagement in health decisions. Its scalable, multiplatform approach offers a replicable model for enhancing citizen participation and equity in healthcare systems worldwide, making it highly relevant for global public health initiatives.

